# Effect of Sertraline on Uremic Pruritus Improvement in ESRD Patients

**DOI:** 10.1155/2012/363901

**Published:** 2012-08-29

**Authors:** Mansor Shakiba, Hoshang Sanadgol, Hamid Reza Azmoude, Mohamad Ali Mashhadi, Hassan Sharifi

**Affiliations:** ^1^Department of Psychiatry, Zahedan University of Medical Sciences, Zahedan 98167-43463, Iran; ^2^Department of Nephrology, Zahedan University of Medical Sciences, Zahedan 98167-43463, Iran; ^3^Department of Internal Medicine, Zahedan University of Medical Sciences, Zahedan 98167-43463, Iran; ^4^Hematology/Oncology Ward, Department of Internal Medicine, Zahedan University of Medical Sciences, Zahedan 98167-43463, Iran; ^5^Medical Surgical Nursing, Departments of Nursing, Iranshahr School of Nursing and Midwifery, Zahedan University of Medical Sciences, Iranshahr 9914786838, Iran

## Abstract

*Background*. Although uremic pruritus is a common and upsetting problem of chronic kidney disease, there is no approved treatment for it. This study was undertaken to find the efficiency of sertraline as a possible treatment for uremic pruritus. *Methods*. 19 ESRD patients under hemodialysis with severe chronic pruritus were randomly selected to participate in this before-after clinical trial. Before and after starting treatment with sertraline, a detailed pruritus history was obtained and pruritus graded by the 30-item inventory of pruritus that patients based on priorities grade allocated to 3 classes. Subjects were treated with sertraline 50 mg oral daily for four months, with monthly assessments of pruritus symptoms. *Results*. Before treatment with sertraline, the grade of pruritus in 9 (47.4%) patients was moderate and severe in 10 (52.6%) patients. After treatment, grade of pruritus in 11 (57.8%) patients was weak, 6 (31.5%) have moderate and only 2 (10.7%) patients have severe pruritus. Of 10 patients with severe pruritus, 5 (50%) patients experiencing weak pruritus, and 4 (40%) patients have moderate pruritus after treatment. Based on Wilcoxon signed-rank test, the difference between the grade of pruritus before and after treatment with sertraline was significant (*P* = 0.001). *Conclusions*. Although no definitive recommendation can be made regarding treatment of uremic pruritus, we found an increased antipruritic effect of sertraline in ESRD patients.

## 1. Introduction

Pruritus, or itch, is defined as a displeasing feeling that irritates the desire to scrape. Certain systemic diseases known to cause pruritus include renal, cholestatic, hematologic, endocrine, and malignancy disorders. Uremic pruritus (UP) is a common and upsetting problem of chronic kidney disease specially end-stage renal disease (ESRD) [1].

Although there are a number of published studies describing the prevalence of UP, the reported prevalence of uremic pruritus in dialysis patients has varied over the years [2]. The global prevalence of moderate or extreme UP among hemodialysis patients is about 42% which was linked with sleep disturbance, depression, impaired quality of life, and mortality [3]. In 2003, Zucker and coworkers published a paper in which they described the prevalence and characterization of uremic pruritus in patients undergoing hemodialysis and demonstrated that the prevalence of uremic pruritus was 66% which was associated with sleep abnormalities in a majority of the patients [4]. Mathur et al. (2010) in their study found that the pruritus were strongly associated with diminished health-related quality of life in multiple domains, including mood, sleep, and social relations [5].

Many factors have been implicated in the pathogenesis of itching in uremic patients. In his review, Lugon (2005) identifies xerosis, high serum levels of Ca, P, Mg, PTH, aluminum, and substance P, elevated cutaneous content of divalent ions, hypervitaminosis A, peripheral neuropathy, the chronic inflammatory condition, and high prevalence of HLA-B35 as contributing factors [1]. Although the level of plasma histamine and the number of mast cells increased in uremic patients, it has been suggested that there is no relationship with the extent of pruritus [6].

Several efforts to alleviate possible contributing factors have been done by delivering adequate dialysis. Regardless of the best attempts at prevention and control, most of the hemodialysis patients continue to suffer from chronic Pruritus [5].

The treatment for pruritus of systemic disease varies depending on the underlying etiology. Recently, a number of treatments used for uremic pruritus such as antihistamines, steroids, emollients, and phototherapy (UVB) have not been investigated strictly, and there is no approved drug for this displeasing problem by Food and Drug Administrations in the world so far. 

Several studies have revealed that the selective serotonin reuptake inhibitors (SSRI) could reduce the severity of pruritus [7–11]. Sertraline hydrochloride is a SSRI that the efficacy of it as a treatment for major depressive disorder was established in several clinical trials. Previously, sertraline was used with cholestatic pruritus which was associated with improvement in itching perception. This view is supported by Browning et al. (2003) whose study showed 86% of subjects who had been given sertraline for another indication improved considerably, and pruritus disappeared in 30% of those individuals [12]. For the first time, Arcoraci and Discépolo (2000) in their study found that sertraline reduced pruritus in 6 patients with end-stage renal disease [13].

In the review of the literature, we found that the research to date has tended to focus on cholestatic priorities rather than uremic pruritus and there is only one study, which cannot adequately cover the effectiveness of sertraline on pruritus alleviation in ESRD patients [13]. Hence, this study was undertaken to find the effect of sertraline on alleviation of uremic pruritus.

## 2. Subjects and Methods

### 2.1. Subjects

19 patients with severe chronic pruritus were randomly selected to participate in this clinical randomized trial. Subjects were recruited from the Nephrology Clinic and Hemodialysis Unit of the Khatam-al-Anbia hospital in Zahedan, Iran and were eligible for the study if they had aged over 18 and chronic pruritus for at least 3 months due to end-stage renal disease (ESRD). 

Subjects were excluded if they were already taking systemic antipruritus one month and local antipruritus 2 weeks prior to study including antidepressant; opioid antagonist; immunosuppressant; capsaicin; cholestyramine; corticosteroids; tacrolimus; UVB phototherapy. Also established primary skin disorder that induce pruritus for example atopic dermatitis and psoriasis; any contraindication to sertraline. Any abnormality (indeed, critical values) in the laboratory tests before treatment including calcium, (normal range: 8.2–10.2 mg/dL), phosphorus (normal range: 3.5–5.5 mg/dL), alkaline phosphatase (normal range: 20–120 IU/L as shown in Table 2), albumin (normal range: 3.2–4.8 g/dL), parathyroid hormone (normal range: 50–330 pg/mL) lead to exclude patients. All patients were dialyzed with bicarbonate hemodialysis liquid 3 times a week. All subjects provided written informed consent prior to study entry. This study was approved and overseen by the Institutional Review Board at Zahedan University of Medical Science. 

### 2.2. Procedure

Before and after starting treatment with sertraline, a detailed pruritus history was obtained at each visit to assess 24 hours pruritus rate, severity, distribution, number and duration of pruritus attack, pruritus induced sleep disorder. Pruritus was graded by the 30-item inventory of pruritus developed by researchers through deep relevant review of literature about pruritus and itching ([1–4, 14, 15]). Committee of expert under observation of Research committee in the Zahedan University of Medical Science, including two dermatologists, two internal medicine, and two nephrologists, assessed content validity of this inventory. The content validity index for this form was 0.82. For the purpose of pruritus measurement, subjects were asked to fill a questionnaire. The reliability of this questionnaire was confirmed by a global retest reliability of 0.84. For each class, sertraline (Sobhan Darou Co., Iran) with 50 mg once daily started and continued for one month. At the end of each month, pruritus was assessed. This protocol was continued for 4 months. At the end, patients were assessed. The patients based on pruritus were allocated to 3 classes: severe, moderate, and weak.

### 2.3. Pruritus Measurement

The questionnaire included the items below:

Note: the number in parenthesis is the point.

1. Pruritus period: one point for each time of the day (morning, afternoon, and night). If the patient suffers from the pruritus all day, give 3 points.
2. Pruritus severity: The severity of pruritus was assessed subjectively and scored as follows: pruritus without the need to scrape (0), limited need to scrape (2), consistent need to scrape (3), scrape without improving (4), and irritant pruritus (5). This pointing includes morning and afternoon, and maximum point was 10.
3. Pruritus distribution: limited to one or two regions for example arm, leg, or trunk (1), generalized pruritus (5), and maximum was point (10).
4. Pruritus frequency and duration: if the duration of pruritus was less than 10 min (1), if more than 10 min (5), and maximum point was 10.
5. Sleeping: the sleeping effect of pruritus was measured by asking individuals whether their pruritus interfered partially, completely, or not at all with certain sleep hours: no night sleep because of pruritus (10) sleep less than 7 hours (10 sleep hours).
6. Waking up: each wake up (1) and wake up more than 5 times (5).

Based on this pointing system, pruritus graded as in Table 1.

### 2.4. Statistical Analysis

Patients were compared according to the variables. Data entry and coding were done and analyzed by SPSS version 18. Wilcoxon Signed-rank test, Student's *t*-test and X^2^ score were used for statistical analysis. Statistical significance was defined as *P* < 0.05.

## 3. Result

The study was performed on 19 patients; eleven males 57.8% and eight females 42.2% with a mean ± SD age of 43.9 ± 12.3 years suffering from uremic pruritus (HD related) with an average duration ± SD of 5.0 ± 4.3 years. Fifteen patients (78.9%) had continuous pruritus all seasons of the year and only 4 patients (21.1%) had pruritus in the warm season. Mean time of hemodialysis was 3.6 ± 3.1 years (range: 1.5 to 10 years). In addition, 2.7% of subjects were suffering from uremic pruritus for about 1.4 years before they started on hemodialysis. Based on the patient self-report and skin examination, of all the patients, 31.5% of them had dry skin. 

Sertraline was well tolerated in all patients without any side effects. None of the patients had psychiatric contraindications for application of sertraline, such as suicide intentions or suicide attempts.

The laboratory tests before treatment with sertraline were near normal range and there were no critical values. Table 2 shows some tests.

**Figure 1:**
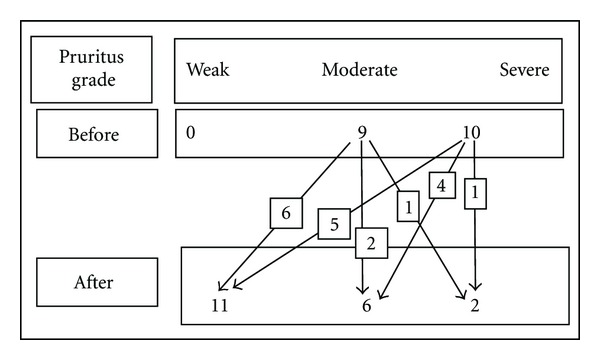
Patients allocation based on the grade of pruritus before and after treatment with sertraline.

**Figure 2:**
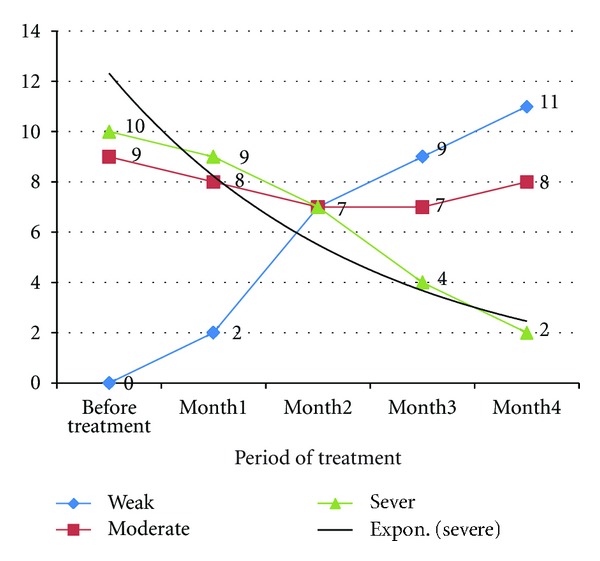
Number of patients with different severity of pruritus during treatment with sertraline.

Before treatment with sertraline, the grade of pruritus in 9 (47.4%) patient was moderate and severe in 10 (52.6%) patients. After treatment, grade of pruritus in 11 (57.8%) patients was weak, 6 (31.5%) have moderate pruritus, and only 2 (10.7%) patients have severe pruritus. Before treatment with sertraline, of 9 patients with moderate pruritus, 66.6% of them experienced weak pruritus after treatment. Sertraline treatment was also associated with a decreased in the number of patients with severe pruritus. As shown in Figure 1, at the end of the 4th month, of 10 patients with severe pruritus, 5 (50%) patients experienced weak pruritus, and 4 (40%) patients have moderate pruritus after treatment, and only one patient has no change.  Table 3 summarized grade of pruritus and Figure 2 shows the number of patients with different severity of pruritus during treatment with sertraline.

Based on Wilcoxon signed-rank test, the difference between the grade of pruritus before and after treatment with sertraline was significant (*P *= 0.001). As depicted in Table 3, result of the Wilcoxon test showed there was a reduction in the rate of patients with moderate and severe pruritus as 67% of patients (*n* = 6) with moderate pruritus before treatment have weak pruritus, 50% (*n* = 5) of all patients with severe pruritus decreased to weak, and 40% (*n* = 4) decreased grade to moderate after treatment.

## 4. Discussion

The present study was designed to determine the effect of sertraline on uremic pruritus in end-stage renal disease. This before-after clinical trial study extends and validates only one previous study that sertraline use is associated with an improvement in uremic pruritus. As mentioned in the introduction, several studies have revealed that the selective serotonin reuptake inhibitors (SSRI) could reduce the severity of pruritus [7–13, 16]. Our finding is in agreement with these studies that showed improvement in the itching perception after treatment with sertraline. To our knowledge, this is the second experimental study investigating the effects of sertraline on pruritus in ESRD patients and showing that pruritus varies significantly before and after treatment. In summary, this drug may be added to the other systemic therapy because it alleviates itching sensation, whereas the evidence shows that in the Lugon review, no definitive recommendation was made regarding treatment of uremic pruritus [1]. 

In the matter of pruritus, maybe the final cure for uremic pruritus is kidney transplantation which some study shows its efficacy. For example, in a dermatologic survey performed on kidney transplant patients the prevalence of pruritus was 10% on compared to ESRD patients which was 60% [1]. Regarding none accessibility of kidney transplantation for all patients, many researchers are interested in conducting study to assess the efficacy of medication for alleviating, preventing, or treatment of uremic pruritus due to ESRD. Herein, some medications reported to be favorable in patients with uremic pruritus. This medication includes antihistamine drugs, activated carbon, cholestyramine, nicergoline, opioid antagonists, a leukotriene inhibitor, erythropoietin, heparin, lidocaine, thalidomide, and fatty acids [1]. Although there is some study that indicated improvement in cholestatic pruritus sensation with sertraline, very little was found in the literature on the question of sertraline efficacy on uremic pruritus. For the first time, Arcoraci and Discépolo (2000) used sertraline in 6 end-stage renal disease patients with the aim of treatment of pruritus and found improvement in the pruritus sensation [13]. This study produced results which corroborate the findings of a great deal of the previous work in this field. We found an increased antipruritic effect of sertraline on ESRD patients when compared to those before treatment. Consequently, this result supports the clinical application of sertraline on patients suffering from pruritus [10–13], nevertheless we cannot prove it due to the study limitation. In this investigation, there are several sources for the note. The main of them is the small size of the sample. Another major source of uncertainty is in the method used to calculate the effect of sertraline on uremic pruritus improvement because we performed a non-placebo-controlled design. 

Based on the great body of knowledge, several elements have been used for evaluating the quality of dialysis including dialysis dose, efficacy of uremic solute removal, and biocompatibility. In addition, some elements such as Kt/V urea, normalized protein catabolic rate (nPCR), and serum level of *β*2MG for evaluating the adequacy of dialysis doses. In the present study, with some considerations, we assessed Kt/V urea, URR (urea reduction rate), BUN, and creatinine for evaluating the adequacy of dialysis dose. Based on the National Kidney and Urologic Diseases guideline, Kt/V above 1.2 and the URR close to 65 percent meet the adequacy of dialysis dose [17]. In the present study, the level of Kt/V was 1.1 and URR was 64% (as indicated in Table 2), which are close to the normal values. However, these numbers and high level of phosphorus (6.7 ± 1.4 (mg/dl)) show that there is a concern about poor dietary or phosphorus binder's adherence; also inadequate doses of dialysis, in return, may restrict the sertraline efficacy. Anyway, with consideration of all of mentioned above, the results have to be interpreted with care and have to be confirmed in future randomized, controlled, double blind studies.

## Figures and Tables

**Table 1:** Pruritus-pointing system.

Item	Morning	Afternoon	Night	Total
Period	1	1	1	3
Severity	5	5	—	10
Distribution	5	5	—	10
Frequency	5	5	—	10
Sleeping	—	—	10	10
Waking up	—	—	5	5

Total	16	16	16	48

Based on the patients sign and symptoms, points allocate to the patients then categorized as mild: 1 to 16; moderate: 17 to 32; severe: 33 to 48.

**Table 2:** The mean ± SD of laboratory tests before and after treatment with sertraline.

Parameter	Before treatment	After treatment	Normal range
Calcium	8.1 ± 1.3 (mg/dL)	8.2 ± 1.5 (mg /dL)	8.2–10.2 mg/dL
Phosphorus	6.8 ± 1.1 (mg/dL)	6.7 ± 1.4 (mg/dL)	3.3–5.5 mg/dL
Alkaline phosphatase	392.6 ± 205.6 (IU/L)	415.7 ± 224.2 (IU/L)	20–120 IU/L
Albumin	4.08 ± 1.4 (g/dL)	3.8 ± 0.6 (g/dL)	3.2–4.8 g/dL
Parathyroid hormone	314.3 ± 199.8 (pg/mL)	301.7 ± 210.5 (pg/mL)	50–330 pg/mL
Hematocrit	31.06 ± 2.9 (%)	32.5 ± 3.4 (%)	M: 42–52% F: 35–47%
BUN	56 ± 12.3	31 ± 4.3	10–20 mg/dL
Cr	7.2 ± 1.2	4.1 ± 1.1	0.7–1.4 mg/dL
URR^1^		64%	>65%
Kt/V^2^		1.1	>1.2

^1^URR: urea reduction ratio: this percent stands for 4-month-period.

^2^Kt/V: Kt stands for dialyzer clearance multiplied by time (mL/min) and V for volume of water a patient's body contains.

**Table 3:** Grade of pruritus before and after treatment.

Before	After					
	Weak		Moderate		Severe	
	Number	%	Number	%	Number	%
Moderate	6	67	2	22	1	11
Severe	5	50	4	40	1	10

Total	11	57	6	32	2	10

Wilcoxon, *Z* = −30343, *P* = 0.001.
